# Thinking with the Rain. The Trajectory of a Metaphor in Vygotsky’s Theoretical Development

**DOI:** 10.1007/s12124-021-09646-4

**Published:** 2021-09-27

**Authors:** Tania Zittoun

**Affiliations:** Institute of Psychology and Education, University of Neuchâtel, FLSH - Tilo Frey 1, CH – 2000 Neuchâtel, Switzerland

**Keywords:** Vygotsky, Metaphor, Rain, Thinking, Affect, Dynamics, Higher psychic functions

## Abstract

*Vygotsky’s Notebooks* edited by Zavershneva and Van der Veer (2018) give us a unique access to the inner-dialogue in which Vygotsky engaged while he was developing his theoretical work. In this paper, I propose to follow as “fil rouge” the semantic field associated to water, rain and clouds, and that will culminate as a “meteorological metaphor” through the *Notebooks*. Following the trajectory of this metaphor enables me to retrace the development of Vygotsky’s ideas about the dynamics uniting the planes of thinking and action, reality and inner life, affects and thinking. Doing so, I hope both to reflect on the role of a metaphor in the theoretical development of Vygotsky’s writings, and to highlight the potential and limits of his last series of theoretical explorations, which may inspire future work.

[The *central problem* of all *psychology*: Freedom].

Lev S. Vygotsky about 1931–1933^1^^,^^2^

Lev S. Vygotsky was a widely read young man, who engaged with the Bible and religious texts, political philosophy, the arts, and then, all the available psychology and educational science of his time. As in the case of other prolific, creative and intensively thinking authors (Zittoun, in press), Vygotsky’s writings are deeply dialogical, mentioning other authors from the past and from his time, arguing systematically, often also supporting his thinking with images borrowed from literature and poetry, form his observations, and from everyday life (for a close analysis see for instance Van der Veer & Zavershneva, 2018). Vygotsky’s analysis of concepts, language, or text, is deeply processual: he was always interested in how things come to be, and how they develop – how the course a poem transforms the reader (Vygotsky, 1971), how imagination transforms the person and society (Vygotsky, 1994, 2004), and how language shapes thinking (Van der Veer & Valsiner, 1993b; Vygotsky, 1934). Elsewhere, we have shown that some of Vygotsky’s ideas may actually reflect his own experience of living intense transitions in a world in transformation (Zittoun & Stenner, 2021). Here, I propose to examine some of the dynamics by which he developed one line of theoretical ideas, by following the trajectory of water.

In Vygotsky’s *Notebooks* edited by Ekaterina Zavershneva and René van der Veer (2018), there is a recurring image: the image of clouds turning into rain, of water into cloud, and eventually chased by wind. At a second look, the image is part of a semantic field related to water, and organised around three main images: that of water as being made by smaller units, which can then be part of wider wholes as the ocean; the water as what extinguishes fire; and water as in the circulation of rain and clouds. Water and its semantic fields appear in Vygotsky’s notebooks with very different functions, in a literal sense or as metaphor, in different contexts – as quotation of a text, or a dialogue with someone. In either case, images related to water enable Vygotsky to pursue an intuition with which he worked through all his career: that of different “levels” or “planes” of psychological functioning. This idea, which Vygotsky explores until the very end of his life, is interesting and important; it both designates core developmental processes, and is related to Vygotsky’s attempt to build a unified model of the thinking, feeling and experiencing person.

In this paper, I thus propose to retrace the evolution of the semantic fields associated to water, which will give rise to a “meteorological metaphor” that plays an important role in the formulation of Vygotsky’s key theoretical ideas. Following the trajectory of a metaphor is based on two theoretical ideas. First, it gives us a “fil rouge” through Vygotsky’s *Notebooks*. Zavershneva and Van der Veer (2018) have done a remarkable work in organising Vygotsky’s notes thematically and chronologically. The notebooks are made of notes Vygotsky wrote for himself – they contain ideas, reflexions, reactions to other’s conferences, quotes, ideas about other’s people’s writings, projects for possible talks and conference, as well as empirical observations. They are also very dialogical: Vygotsky reasons, asks questions, argues, objects, punctuating his inner dialogue with Latin rhetorical terms. The notebooks are thus part of Vygotsky inner-dialogue; not strictly a diary, they nevertheless constitute a form of self-writing. Such dialogical self-writings are part of one’s self development: they enable its author to put ideas into words, to reflect upon them, to display one’s inner dialogues reflecting dialogues with other – it supports the elaboration of one’s experiences and theoretical ideas (Arendt, 2005; Gillespie & Zittoun, 2010; Lejeune, 1998; Zittoun & Gillespie, 2012, 2021). Hence, following one recurrent theme enables me to traverse the notebooks, and to retrace the evolution of Vygotsky’s thinking over twenty years, from his first interests to his final interrogations. Second, the theme of the water becomes a theoretical metaphor in Vygotsky’s writings. It is admitted that metaphors, and especially live metaphors (Ricoeur, 1997), are semiotic resources for thinking, usually anchored into embodied experience, which enable to reach abstract ideas (Arendt, 1978); in turn, metaphors shape the theories and models they constitute (Campill & Valsiner, 2021; Cornejo, 2010; Zittoun & Gillespie, 2020). Following the development of a metaphor, I propose, enables us to retrace the evolution of Vygotsky’s theoretical thinking. Following these two lines of reflection, my central argument is that Vygotsky’s metaphorical thinking around water became a core resource to develop his theoretical understanding of the dynamics uniting two planes of consciousness. As corollary, I also suggest that Vygotsky’s theory of the development of thinking reflects his own experience of metaphorical thinking. Finally, this exploration brings me to highlight questions that Vygotsky left open and that may inspire future work.

Methodologically, I identified all the mentions of water, rain, and clouds in Vygotsky’s *Notebooks*, and tried to understand the context of these mentions. In parallel, as I realised that the apex of the use of the metaphor is related to Vygotsky’s formulation of the dynamics uniting two planes of consciousness, I also try to retrace the main steps of his exploration of two levels of consciousness. I made the choice to present most quotes evoking water and its semantic fields, and some quotes reflecting the more general evolution of Vygotsky’s ideas across the periods identified by Zavershneva and Van der Veer (2018). I chose to present this trajectory using the present time. In all the quotes, italics and brackets are those proposed by the editors of the *Notebooks*.

## Natural Events - Pre-theoretical Mentions

Through the *Notebooks*, there is a first series of occurrences of water and rain, used in the literal sense. The first three chapters edited by Zavershneva and Van der Veer group Vygotsky’s youth reflexions on the Bible and Israel. There, the themes of water and rain appear as Vygotsky quotes such texts: in 1912, The Ecclesiastes, where “the king of Israel (…) made gardens (.), made cisterns of water to water the gardens” (p.3). Later, in 1916–1917, Vygotsky discussed and rejects the possibility of “Jewish politics”, which, “sound like ‘dry water’ and a ‘round square’, a contradiction in adjecto” (p. 31) – a case in which water is used in a logical analogy. Once Vygotsky moves to discuss poetry and “genres of writing” as preparation for *the Psychology of Art* (Chap. 5), (around 1923?), he starts reflecting on the metaphorical uses of notions including water. Vygotsky reflects on the role of images in different genres of writing, such as images in lyrics “on the ethereal ocean” (quoted from Lermontov, note 5 p. 34), or in myths:

The difference between the image and the myth is only in our attitude: whether we believe in its reality or know that it is just poetry. For example: (…) a bleeding heart, a broken heart (…) Glinskaya took the human heart, put it in water, sprinkled it riding through Moscow, Moscow burned. (Vygotsky in Zavershneva & Van der Veer, 2018, p. 52)

This anecdotic, but powerful image of Moscow burning, and of what sorts of water one needs to extinguish such a fire, is to be noted. Zavershneva and Van der Veer present the events to which Vygotsky refers; they explain that Moscow burned three times in 1547, and that according to the legend, the boyars asked the assembled crowd who set the fire; the crowd answered: “’The Glinskiy’s’; (…) it was said that their mother, princess Anna, took the hearts from the dead and put them in water, which she then used to besprinkle the streets of Moscow” (*Encyclopedian Dictionary*, 1983, entry 865–866, quoted by Zavershneva & Van der Veer, 2018, p. 55, note 10). For reasons that we ignored – but perhaps Vygotsky has seen such fires? – the image seems to have impressed him, and as we will see, images of fires and water-hoses will be recurrent in the *Notebooks.* Also, this indicates Vygotsky reflection on the role of images that can be used “for poetry” without believing in them, that is, his reflexion on the role of metaphorical or poetic thinking (Zittoun & Stenner, 2021).

In 1925, Vygotsky travels to Europe, his only trip out of the USSR, to attend a conference in London. This trip made him a strong impression (Van der Veer & Zavershneva, 2011) ; it is also one of the only times that Vygotsky mentions the actual weather, together with the places he visits: August 1st, he mentions Westminster Abbey; August 4, Vygotsky reads the whole day, and comments “Rain all day” (Vygotsky in Zavershneva & Van der Veer, 2018, p. 61).

## *As Clouds into Rain…* The Trajectory of a Metaphor

Soon after this trip, Vygotsky spends half a year in 1926 at the hospital, and he intensively develops his ideas during that period – on the role pf psychology^3^, on meaning, *Hamlet*, and his psychology of arts. He develops here his reflexion on the two planes a novel can create – between plot (material) and subject (form) (Vygotsky, 1971; Zittoun & Stenner, 2021), on the dynamic uniting them, and on how contrasting lines can lead the reader to emotional abreactions or transformations. He also reflects on the relation between dialectical materialism and biological science and psychology, and whether the former can be applied to the latter; he reflects on the role of “Hegel’s *examples*. But there the goal is to clarify the idea, but here? Water-ice-steam and subsistence economy – feudalism – capitalism from the viewpoint of dialectical materialism is *one and the same*, but for historical materialism – what a *qualitative* wealth in feudalism-capitalism!” (Vygotsky in Zavershneva & Van der Veer, 2018, p. 88). This reflection seems interesting in the evolution I wish to retrace for two reasons: first, because Vygotsky is still trying to reflect on the function and power of analogical or metaphorical thinking; and second, because the image used to reflect on historical development is that of the transformative continuum of water, from ice to steam – an image already implicit in earlier metaphors, but that will be recurrent later on. Here, it starts to be applied to psychology in a series of reflections on mind, in which consciousness is compared to a stream – as in a river, as in James?:

Mind is the formation of something stable amidst the streaming. This is its positive role – it is not about reflection – the non-mental also reflects (the thermometer), but about incorrect, i.e., subjective reflection. To distort reality to the advantage of the organism.

If we would see all things, all changes, all properties of the drop of the water in the microscope, and a river? But the selection of really higher forms is achieved in mind. The red, the blue, the loud – we cut the world into portions so that I can eat it and not break my teeth (Vygotsky in Zavershneva & Van der Veer, 2018, p. 92).

Mind is thus what decomposes the stream of experience into ways which are manageable for the person; it decomposes into portions - we cannot see the river in the drop of water – but the diverse is then put back together in mind. We do not reflect the world; we recreate it for our use.

As time passes, between 1927 and 1929, Vygotsky is defining his instrumental psychology; he is still preoccupied with the definition of psychology – “Psychology is the science of mental life. But what is mental life? The answer is psychology as a whole” (Vygotsky in Zavershneva & Van der Veer, 2018, p. 109). Yet within this whole he is distinguishing lower from higher functions, which he tries to qualify, and he is in search of a name and “a doctrine contained in this phrase: the *historical* theory of the *higher* psychological functions” (Vygotsky in Van der Veer & Zavershneva, 2011, p. 122). Between 1929 and 1931, Vygotsky develops a systemic principle in his understanding of the development of concepts, and in 1931 there is a note on the anomalous development of the child. He then pursues his explorations of emotions, meaning, sign and language, notably through his descriptions of children at the EDI clinic^4^. Vygotsky describes the social conditions of their upbringings, their conduct, commitment to learning, attention to other children, etc., and makes hypothesis about how they “compensate” for domains they have difficulty with. Although he does not fully develop this point, it however suggests that children may engage differently in different planes of activity^5^. Emotions are then further explored in link with Spinoza; here Vygotsky reflects on the link between organic and psychic aspects in emotions, maintain a debate with the James-Lange question, in dialogue with classical philosophy. “Socrates sat in prison because the muscles of his legs contracted and led him there. Socrates calmly drank the poison cup because of the peristalsis of his intestine, etc. *Cause and effect in one plane.* (…) The question of the [relationship between] the emotions and the organs is the question [as to whether] the eternal laws of nature or the historical laws govern the emotions?” (Vygotsky in Zavershneva & Van der Veer, 2018, p. 211). Vygotsky seems to find a solution around 1932, where he formulates the idea that there are “higher emotions” such as the one enabling freedom and free will. He writes a draft for a plan for an unwritten text on theory of emotions (Vygotsky in Zavershneva & Van der Veer, 2018, p. 222), and it is worth to reproduce this:

1. Knowledge has levels (concepts).
2. The affect has levels (the [level of the] concept corresponds with [the level of] the affect).
3. Freedom has levels (the [level of the] concept corresponds with [the level of] freedom).
4. My study. Consciousness has a semantic and a systemic structure.
5. Height psychology. *Ecce homo*.

NB! But this higher is not given from the very beginning. It must be achieved with difficulty (…) *Ergo*, the task to *prove how the higher is possible in man* requires the motion from lower to higher, it requires development. (Vygotsky in Zavershneva & Van der Veer, 2018, p. 224)

The points will remain central in Vygotsky’s future work: how do we move from the lower to the higher levels or planes, how do we connect organic and historic? How do affects transform, how does meaning operate through language and sign, and how to account for a consciousness which is both systemically organised, yet streaming like a flow?

## Rain, Clouds and Wind – a Core Theoretical Metaphor

Around 1932, Vygotsky explores these ideas notably in relationship with his preparatory work for *Thinking and speech;* at that time comes an abondance of metaphors related to water and clouds in the *Notebooks*. Vygotsky writes a series of notes after internal conferences and as objections to his colleagues, and he is trying to catch the relationship between meaning and consciousness (“Meaning in the psychological sense is the internal structure of the sign operation. The sign mediates through meaning. We understood it as an aspect of behavior: we must understand it as an aspect of consciousness” (Vygotsky in Zavershneva & Van der Veer, 2018, p. 259)). Here comes an interesting passage:

52. “The clock fell”. “The clock fell”: (1) He wants to explain why the clock stopped: (2) he wants to explain why this sound or noise of something falling was heard, i.e., an answer to two different questions. The fact that the semic and phasic sides of speech do not coincide is the first that the analyses establishes. Secondly: the thought and the meanings do not coincide. Behind this is the thought: I am not guilty. The same thought could be expressed as: I am not in the habit of touching other people’s things, I was dusting, etc. All these meanings mediate the thought, which without the word is incorporeal and a Stygian shadow (Mandel’shtam) – the vague wish to justify oneself, and perhaps, thought most of all contains all these mediations as a cloud. (…) The whole point is that direct communication between one consciousness and another consciousness (…) is impossible (….). But without the word, we ourselves do not understand our thought – when ‘the word remains unconscious’. (Vygotsky in Zavershneva & Van der Veer, 2018, pp. 259–260).

I quoted this passage extensively because this may be, according to the Zavershneva and Van der Veer, “one of the first uses of the ‘wind-cloud-rain’ metaphor, which stand for motivation, thought and speech and figures in the last chapter of *Thinking and Speech*” (2018, n. 38, p. 267). As we saw, it may be the first time the meteorological metaphor is used to designate the relationship between consciousness and thought, and forms of externalisation, but not the first time it is used by Vygotsky. As retraced so far, it already went through various layers of transformation and possible connotations.

From this point on, there is one key period during which Vygotsky intensively refers to the meteorological metaphor, in December 1932. A series of notes seem to be related to a symposium on December 4th, 1932, in which Vygotsky reflects on the systemic relationship between lower and higher functions, motivation, thinking and action, through development and the mastery of language and double stimulation. He writes:

The cloud above speech: also a cloud above the action. To do research via action is as complex as via speech.

*Questions*: (a) *word meaning and the concept*, do they blend at the end of the road, but not along the road; (b) form and content; (c) the cloud of motivation (the wind)-except word meanings; the question of *volition-motivation*. (Vygotsky in Zavershneva & Van der Veer, 2018, p. 277)

And a bit later the metaphor becomes focused on the relationship between language and speech – this is the full development of the metaphor:

(…) The *cloud* is thinking? Or becomes thinking when it pours into speech. (…). *This is the problem of thinking and speech*. (…)

2. If the *thought* is a cloud that pours a *shower* of speech, then (1) the clouds have their movements, their raindrops have their own, although they are connected: The rain moves with the cloud, the cloud sheds rain, etc. The speech motives are the *wind* that brings the cloud into movement: It has its own movement, which is also connected. There are clouds that do not pour a shower (Uspensky’s petitioner); there is powerless wind and other wind that dispels the clouds and does not gather them.

3. We do not distinguish sign and meaning in order to return to the idea that speech is the garment of though. Everything is full of distinctions and transitions. (Vygotsky in Zavershneva & Van der Veer, 2018, p. 278)

**Fig. 1:**
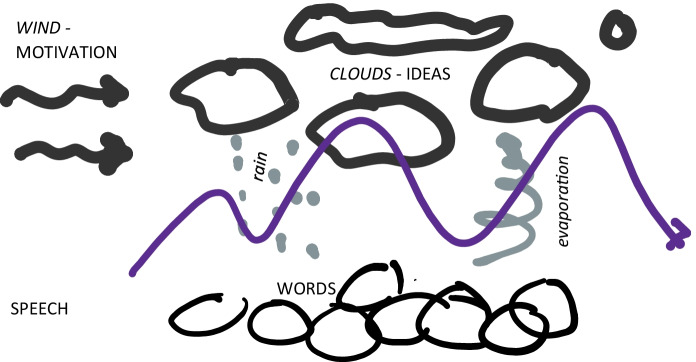
The meteorological metaphor

In the full deployment of the metaphor, Vygotsky is exploring the relationship between motives or motivation, thinking and language – wind, clouds and rain. The metaphor is highly instructive because it implies a dynamic force, a drive, a motivation that in some ways animates thinking. Yet clouds have different properties than the wind, so the movement communicated by motives may result in an idea taking shape, or dissolving shapeless; and under some conditions, some thinking is transformed and decomposed into the units that are rain drops, and language that has its own dynamic. Language is never a pure expression of thought; thought can be transformed by speech; all are supported by motivation that could not be perceived itself (see Fig. 1). Note that through his exploration of the meteorological metaphor, Vygotsky is also trying to articulate a complex phenomenon; his ideas about the relationship between motivation, thinking and speech seem to evolve as he explores the ramification of this living metaphor, which itself shapes his thinking (Leary, 1994; Levinas, 2012).

The metaphor comes back in a later entry, still during the same period, this time on the study of consciousness.

*The problem of inner speech – its completely special function*: *Ergo*, it is a neo-formation of *central* interest to us. For it is the *transition* of an external, mediated question into an internal one, i.e., it is the prototype of all historically formed functions. In a certain sense, *it is opposite to external speech* (.). No: External speech is the process of transforming a thought into words, its materialization and objectivation; *what the direction concerns, here, here we have* the reverse process – *from outside inward*, the process of *evaporation of speech in thought* (there [it was] rain from cloud, i.e., the steam of mind turns into material liquid). (…) Consciousness does not completely evaporate and does not disappear in pure spirit. But whereas in external speech the thought becomes embodied in the word, in inner speech the word *dies* and gives birth to the thought: thought by *pure meanings*. “Strangers to the sky soon tire”. Thought and word in inner and external speech move in opposite directions. (Vygotsky in Zavershneva & Van der Veer, 2018, p. 283).

Here Vygotsky pursues the exploration of the “transitions” between different states of matter in the metaphor, and identifies two opposite dynamics: evaporation, by which language liberates its meaning into thought or the “steam of mind”, and materialisation, or embodiment, and objectivation, by which the thought becomes visible into words. Evaporation is the process by which “historical functions” are created, that is, result from cultural dynamics. Finally, Vygotsky has this strange reflection on the highest forms of this evaporation; can thought disappear into pure spirit? No, rather it seems that once words have led to thought, they can dissolve and disappear – they have no place at the atmospheric level of mind. This is the idea expressed through another metaphor, this time via the use of a symbolic resource, a poem by Tyutchev, ““The gleam” (1824 or 1825): “Strangers to the sky soon tire/We are common dust/We cannot breathe such fire”” (Zavershneva & Van der Veer, 2018, p. 289, note 34).

In a series of notes probably meant for a talk, Vygotsky pursues the implication of this way of conceptualising mind; he thus proposes not a depth, “but height psychology” (Vygotsky in Zavershneva & Van der Veer, 2018, p. 285) and explores some of its implications. “Is everything in consciousness meaningful? No. It is dynamic. Not everything is bright. But the light shineth in darkness; and the darkness comprehended is not” (Vygotsky in Zavershneva & Van der Veer, 2018, p. 285). This line of reflection examines the relationship between thinking and consciousness, and the manner in which one becomes aware of the meaning of one’s thinking; “*Thoughts change when we become consciously aware of them*” (Vygotsky in Zavershneva & Van der Veer, 2018, p. 285). Vygotsky explores this in relation to his knowledge of clinical cases: in schizophrenia, “there is a sort of delirium (…) The non-correspondence of the meanings with their real role in consciousness” – and the schizophrenic does not understand himself (Vygotsky in Zavershneva & Van der Veer, 2018, p. 285).

This possible disconnection between words, meaning and thinking has a theoretical implication that is once again explored through the meteorological metaphor:

21. *Most important is: Word meaning* and *thought* do not coincide in consciousness. Meaning is the *path from word to thought* and *from thought to word*. I have a thought: a cloud is hanging above my speech, which sheds it like rain. Thought is reorganised *in meaning* – Thereby *the word* is found. Thus, in the thinking-speech problem, meaning is *part of the word* and not part of thinking, i.e., it lies *in the sphere of speech*. (Vygotsky in Zavershneva & Van der Veer, 2018, p. 286)

In the notes than follow, Vygotsky now uses psychological concepts: how the 4–5 year old child can, with the acquisition of language, start to plan action thanks to intellectualisation, the “path to concepts”; how transfer is one aspect of thinking, as “*Thinking: old experience in a new situation*” (Vygotsky in Zavershneva & Van der Veer, 2018, p. 286). Thus generalization proceeds at the expense of memory, and the concept, that enables this transfer, demands “to lose something in order to find something” (Vygotsky in Zavershneva & Van der Veer, 2018, p. 287). However, only with generalization and abstraction, that is, loosing the specificity of a situation, is there consciousness: “Without abstraction, there is no conscious awareness of the experience that overwhelms us. Cf poetry. This is the meaning of one’s experience” (Vygotsky in Zavershneva & Van der Veer, 2018, p. 287).

Still in 1932, Vygotsky writes a series of notes related to “the semic method” which was meant to become the method to study mind and consciousness, according to Zavershneva and Van der Veer (2018, p. 291). In these notes, the meteorological metaphor comes back in a text presumably also written in December:

Inner speech gives *the same* to speech as speech gives to external action, i.e., as new field and a new “system of freedom” in the speech field. (…) Cf. during a long (external) speech inside me there is its theme, its titles, simultaneously *merged* in a single word (the influence of the senses), thoughts, clouds above the rain; there is an < illegible > inner speech field. *Inde*, the phenomena of *Leeres Sprechen* and *Automatensprechen*. (…) Inner speech is a new flight from speech, a new level of abstraction (Vygotsky in Zavershneva & Van der Veer, 2018, p. 292).

In this passage, the meteorological metaphor is used to reflect upon inner speech, a reflection which seems based on Vygotsky’s phenomenological experience as orator (i.e., “during a long speech inside me”). In the metaphor, clouds now stand for inner speech; as cloud can be distinct from rain, inner speech constitutes a “system of freedom”, distinct from speech and discourses, an inner space not perceived by others (a form of interiority?). This would also explain why some develop an “empty speech”, when it is disconnected from such inner speech (although it is hard to imagine a rain without clouds?). Zavershneva and Van der Veer note that these elements will be later reformulated in the last chapter of *Thinking and speech* (2018, p. 303, notes 3 and 4), a point they systematically explored (Van der Veer & Zavershneva, 2018). Here, in the context of the notes on semic analysis, Vygotsky develops these ideas in dialogue with others – objecting to Piaget and Lévy-Bruhl. It is worth noting that his objection to Piaget is first formulated through a liquid metaphor, before being reformulated in theoretical terms:

*Piaget* has no theory of development. A container with red water, which is cleaned with a stream of white water; a pink liquid that becomes whiter and whiter. (…) Piaget’s postulate: words are immutable, the operation changes; a hidden associationism. (Vygotsky in Zavershneva & Van der Veer, 2018, p. 292).

Again, Vygotsky catches his theoretical intuition through a visible, tactile and dynamic metaphor (red water is replaced by white water in a container, holds for the transformation of operation in Piaget’s structure theory) before formulating it in conceptual terms. He thus reads Piaget as ignoring the construction of words and concepts in his analysis of the development of operation; in contrast, he argues that concepts themselves are dynamic. He suggests that “for the psychologist, the concept is movement. The psychologist and the logician study various forms or types of the movement of the concept”. Indeed, the psychologist studies the movement in human consciousness, disregarding the system of objective knowledge, while the logician does the opposite, “disregarding *who is thinking”.* Consequently, “Lévy-Bruhl and Piaget, in particular, mix up logic and psychology” (Vygotsky in Zavershneva & Van der Veer, 2018, p. 293).

In contrast, Vygotsky examines the person who thinks, and in these notes, he puts also his theoretical ideas in dialogue with his empirical knowledge of children from the clinic in which he works and of his own daughter, Asya, aged a bit under 2 in 1932. Let me mention part of the series of the entries about Asya, as they illustrate the idea that concepts are movements, and are illustrated with the meteorological metaphor. First, Vygotsky writes about play, which is “an abstraction of the action from the concrete field. In Asya it is absent” (Vygotsky in Zavershneva & Van der Veer, 2018, p. 294). Then there is a long entry about her use of “pu-fu”, which is “an example of a complex” and can designate a bottle, iodine, a bruise, etc.; used as one-word sentence, it can be used in different ways, with different objects (give the bottle, the doll hurts herself, etc.). Then this becomes used in interactions, and it is Vygotsky’s reaction to pu-fu that seems to confer its meaning (“Pu-fu! – Give? – Yeees”). Yet this differs from Asya’s capacity to name the parts of the body of a Buddha in front of which she sits. Vygotsky thus comments that in this case, “word meaning is realized in the literal sense (…), meaning is thrown by her finger to the body parts” (Vygotsky in Zavershneva & Van der Veer, 2018, p. 298). Then he wonders:

*Our* problem: Why does Asya, while seeing the whole, in speech mentions the parts. Thinking contains the whole Buddha (her conversation topic, her speech intention; it is not a reflex chain, she demands to place Buddha in front of her and foresees the *whole* activity – compare: A cloud [is a] thought, a shower of words. (Vygotsky in Zavershneva & Van der Veer, 2018, p. 298)

So Vygotsky wonders, how can we explain that Asya seems to experience and probably think the Buddha as a “whole” – she has the cloud -, when she can only express such experience into fractions through the words she masters?

In what follows in this portion of the *Notebooks*, Vygotsky then reflects in theoretical terms about the relation between the motive of thinking, meaning, sense and sign, developmentally, then in discourse, play and poetry.

## The Decomposition of Water

After 1932, there are almost no mentions of the meteorological metaphor in the *Notebooks.* There, Vygotsky continues to develop his reflection in dialogue with other authors such as Freud and Lewin - whom he had met-, and his analysis of children and adults with difficulties. There is one use of water as metaphor to reflect on epistemological issues in 1933:

From the physical viewpoint, the resolving of water into H_2_ and O_2_ is not at all analysis but ascent to the general: As a result of the “analysis”, we get more and not less than [exists] in the object of study: H_2_O is suited both for the Pacific and the raindrop – It is about water in general and not about its individual properties (the extinguishing of fire, Archimedes’ law). The same in pedology: To say about a concrete particular issue (character) that it [results] from environment and heredity means not to break down into its component parts (it is not an analysis). (Vygotsky in Zavershneva & Van der Veer, 2018, p. 396)

As water cannot be separated in its components, development cannot be explained by isolating environment from genetic forces; and the core explanatory principle applies at all scales, from raindrop to ocean. Note here the mention of extinguishing a fire, reminiscing the Moscow fire….

Then there are also a few mentions of water in metaphorical or literal sense, in a series of example pertaining to Vygotsky usual semantic field: looking for the link between communication and meaning he mentions “the carafe with water” (Vygotsky in Zavershneva & Van der Veer, 2018, p. 357); in his presentation of the case of Mikhail P., a difficult child, Vygotsky mentions his love for water-melons which he uses to steal and the novels he reads (Vygotsky in Zavershneva & Van der Veer, 2018, p. 439); later he mentions children’s play which, when complex, “does not need additional material (except for water for the firefighters)” (Vygotsky in Zavershneva & Van der Veer, 2018, p. 471) – again a matter of fire.

In parallel to the *Notebooks*, *Thinking and speech* will be completed in 1934. There, most of the previous water and meteorological metaphors will be reproduced. In the first chapter, the problem of thinking and speech and its unity is presented by using the metaphor from 1932 of the decomposition of water into its constitutive elements:

When one approaches the problem of thinking and speech by decomposing it into its elements, one adopts the strategy of the man who resorts to the decomposition of water into hydrogen and oxygen in his search for a scientific explanation of the characteristics of water, its capacity to extinguish fire or its conformity to Archimedes law for example. (Vygotsky, 1934, p. 43)

The last chapter of *Thinking and speech* then strangely uses most of the metaphors developed earlier. Mecacci has recently shown that Vygotsky uses the analogy of a droplet of water able to reflect the sun, an analogy already mentioned in 1926 by the author as he wrote on the crisis in psychology, and that was borrowed to a poet and philosopher, Vjačeslav I. Ivanov (Mecacci, 2016). Then, the other metaphors pertain to notes from 1932 (see also Van der Veer & Zavershneva, 2018). The problem of decomposition of water is restated in Chap. 7 (“To say that water consists of hydrogen and oxygen is to say nothing that relates to water generally or to all its characteristics. It is to say nothing that relates to the great oceans and to a drop of rain, to water’s capacity to extinguish fire and to Archimedes’ law” (Vygotsky, 1934, p. 246)). More importantly, the whole proposition of the relationship between motivation, inner speech, thinking and speech is developed through the meteorological metaphor, starting with the example of the orator:

Thought is always something whole, something with significantly greater extent and volume than the individual word. Over the course of several minutes, an orator frequently develops the same thought. This thought is contained in his mind as a whole. It does not arise step by step through separate units in the way that his speech develops. What is contained simultaneously in thought unfolds sequentially in speech. Thought can be compared to a hovering cloud which gushes a shower of words (Vygotsky, 1934, p. 280).

And ending with the final proposition on motivation as a wind:

Thought has its origins in the motivating sphere of consciousness, a sphere that includes our inclinations and needs, our interests and impulses, and our affect and emotion. The affective and volitional tendency stands behind thought. Only here do we find the answer to the final “why” in the analysis of thinking. We have compared thought to a hovering cloud that gushes a shower of words. To extend this analogy, we must compare the motivation of thought to the wind that puts the cloud in motion. (Vygotsky, 1934, p. 281)

It is surprising that this powerful metaphor only comes in Chap. 7, when its strength and importance in the *Notebook*s seem much greater. It is beyond my knowledge to explain why it only comes there: is it an effect of the precipitated editorial work by Vygotsky’s team (Van der Veer & Zavershneva, 2018)? Was it Vygotsky’s deliberate intention to keep it for the last chapter? Or is this due to the fact that by 1934, Vygotsky had now theoretically integrated the ideas initially developed through the metaphor, and did not need it anymore – once disinvested and re-theorised, the metaphor fell back to the limbo (Gillespie & Zittoun, 2016; Winnicott, 1959; Zittoun, 2010)?

## *Lighting a Fire* – New Theoretical Explorations

In any case, let us come back to the *Notebooks.* Actually, during the final years of his life, Vygotsky’s theoretical interest has moved away from language and thought, to the more fundamental and underlying issues of the relationship between affects and thinking, and between thinking that is conscious, and that which is not. He thus writes:

We followed a path opposite to that of Freud (he [began with] the theory of the unconscious, we [with] the schema of consciousness), [but] arrived at the same problem. The different paths must make themselves felt (we arrived enriched at the same point), but neither can we begin our study from scratch (Vygotsky in Zavershneva & Van der Veer, 2018, p. 394).

As part of this line of reflection, the theme of water and fire comes back in the last series of notes written by Vygotsky in 1934 which concern his observations of patients, and especially patients Z. and K. Patient Z was a 54-years old woman working as dentist who started to show signs of forgetfulness and confusion from her 40s, and started confabulating about an imaginary child. Patient K was a successful dentist aged 51 who started to suffer from headache aged 43 and progressively became unable to take care of himself (Zavershneva & Van der Veer, 2018, p. 483). Both suffered from Pick’s disease, a frontotemporal dementia that has as symptoms difficulty in language and aphasia, and behavioural changes (Zavershneva & Van der Veer, 2018, p. 490). The interesting point is that K. and Z. seemed to constitute two opposite cases; as Zavershneva and Van der Veer explain,

K’s behaviour was heavily constrained by the concrete external field, and he no longer had the capacity to “stand above the situation”, whereas Z. seemed to have lost almost all contact with external reality and appeared to be at the mercy of her own emotions and motives. Neither was able to flexibly switch from the plane of irreality (thought, dreams, emotions, fantasies) the plane of reality and back again (Zavershneva & Van der Veer, 2018, p. 483).

Vygotsky reported the alteration of language that followed patient K’s illness. I report here his description: interestingly, the sentence he proposes K. to test his understanding pertain to the usual water-fire semantic field:

[Patient] K. (…)

*Speech.* The repetition of words and sentences. He does not repeat [but] associates (picture-portrait, Germany – Wilhelm). (…). When repeating one phrase, it falls apart. The rephrasing (“somewhere near the raven he found a piece of cheese”). *Clouds gathered in the sky* and it started raining: two words. He retains the content, but not the words – glass – *inde* the rephrasing. Fire extinguishes water – *fire*… smothers water. Water extinguishes water – *of course not.* He understands it but cannot say it. Fire is extinguished with a hose, but what is in the hose he cannot say – fire extinguisher.

What extinguishes a fire? Yes (after a short story about firefighters). Fire extinguishes water? Silence. The identification of meaning in different forms. A complete disintegration of the understanding of the phrase “water extinguishes fire”. He is not able to judge what is right and what is wrong. With a nozzle. The stories with “fire extinguishes water” and the other way around. (…) (Vygotsky in Zavershneva & Van der Veer, 2018, p. 491).

Two points can be made. First, strangely, the material Vygotsky uses to test K. seems related to the texts that inspired him in the early 20 s: one of Krylov’s fables explored in the *Psychology of art* (1971), and also, the vision of fire being extinguished, that seemed to echo the first mentioned of the Moscow fire ten years earlier. Second, the observations Vygotsky reports will play a key role in the last steps of his reflection, now examining the relationships between the plane of thought and an anchorage in reality which may, or may not be connected through words.

The editors of the *Notebooks* group a last series of notes under the name “The last conference (patients Z. and K.) or: *Pro domo sua*” (Zavershneva & Van der Veer, 2018, pp. 494–497). These are triply important in the frame of this paper: first, because this line of thought is catalysed around the old semantic field of water; second, as we will see, because the final attempt to theorise the phenomena is made through the same line of water-related metaphors; and third, because it constitutes the theme of the very last entries of Vygotsky’s notebooks. The notes start with “The conference” written in pencil, then some observations of K. and Z. are reported. Of K., Vygotsky writes:

*K.* His memory is disturbed because of the absence of meanings – [what is preserved is] just the object relatedness. But the body memory [cf. the *Wiederaufnahme*). Memory requires the transition from one situation to another one: the real to the semantic field. When the semantic field and the meaning perish, *there must be amnesia.* (…) [The *Starrheit* of the dynamics precluded the dynamics of the semantic fields in K. because there is no fluidity]. (Vygotsky in Zavershneva & Van der Veer, 2018, p. 495).

Here Vygotsky introduces a new idea: that of the dynamics of mind being likely to acquire “*Starrheit*”, rigidity, or to come to a halt. This tension between fluidity and rigidity of consciousness, visible in pathology, can be seen as extension of his water metaphor; it certainly pertains to other psychologists of his time (for instance in Freud); it may open new roads for explanation.

A bit below, Vygotsky writes about Z: “*In* Z, words are an *Ersatz* action: [caused] by the isolation of meanings from the object relatedness” (Vygotsky in Zavershneva & Van der Veer, 2018, p. 495). Then Vygotsky comments, first briefly in the margin “[Here (K.) [there is activity without actions; in Z., there is fantasy without *activity*]”, then announced by an encircled “sehr wichtig”, the following explanation:

The unity of affect and intellect (*correct*) – to *find the unit.* But apparently fluid dynamics do to not *at all* exist *outside thinking* (this is the essence *of the unity*); ergo, insofar as they are found in the dynamics of the field, *they are introduced there from thinking*. *Not like this*: There exist dynamics of two sorts (fluid, free and *starre*, constrained) independent of the intellect (the elements: hydrogen), and there exist two sorts of activity (thinking and real activity) independent of the dynamics, and these two sorts of dynamics can mix in different combinations (oxygen and hydrogen). *But like this*: There exist two unities of *dynamic activity*: thinking and real activity. Both have their *dynamic aspect*, i.e., there is a dynamic system *sui generis* of a specific type and sort. Outside activity, the *two types of dynamics* do not exist *in abstracto. This is the most important and fundamental.*

*Ergo*, we can say that the disturbance of *thinking is primary* (understanding thinking as a *dynamic-semantic system*, as mental life). *Inde the dynamics of the specific sort that correspond with thinking disappear everywhere* and gives way to the *pure dynamics of the field.* (…). *Both these pure cases* exist in K. and Z., because in one there is absolutely meaningless action, and in the other there is absolutely unreal thinking. (Vygotsky in Zavershneva & Van der Veer, 2018, p. 496)

Hence, Vygotsky is extending his system: there are the planes of action and thinking, and dynamics which can be fluid or rigid; through these fluid vs. rigid dynamics, the two planes can enter in dynamic relationships, or not. Meaning seems to emerge when these dynamics are fluid enough. The next few entries continue on this line, exploring “the unity of thinking as an activity implying fluid dynamics” (Vygotsky in Zavershneva & Van der Veer, 2018, p. 497), and in link to the cases of Z. and K: in K. dynamics of the field are preserved, in Z some dynamics of thinking are so, and Z keeps remembering the birth where there is a form of dynamic affective memory. Vygotsky now adds affects: in Z. and K., there are two types of affectivity: “with her – the dynamics of thinking (the second sort). With him – the dynamics of the real field (the first sort)”. Hence, “In K., the piece of fantasy is damaged (the first sharp bend of the zigzag); in Z., it is hypertrophied (there is no second bend – [the analogy in Lenin:] idealism, religion” (Vygotsky in Zavershneva & Van der Veer, 2018, p. 497)^6^.

It is just after these considerations that Vygotsky ends up with the very dramatic last entry: “*NB! Pro domo sua*. This is the last thing I have done in psychology, and I will die at the summit like Moses, having glimpsed the promised land but without setting foot in it” before closing on the Shakespearian “The rest is silence” (Vygotsky in Zavershneva & Van der Veer, 2018, p. 497).

**Fig. 2:**
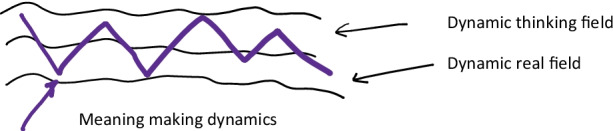
Two planes model of psyche

**Fig. 3:**
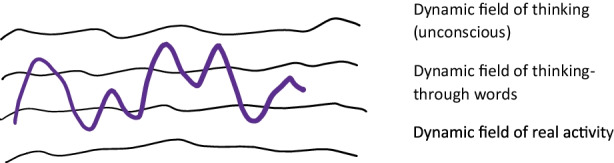
Three-level planes model of psyche

How shall we understand these last pages? In my understanding, Vygotsky uses the two cases of Z. and K. as extreme cases to reflect on the relationships between affect and thinking, and thinking and activity. It seems to me that, in continuation with his earlier reflection through the meteorological metaphor, Vygotsky considers human activity as occurring at two dynamic planes, or fields, mutually also dynamically related: the dynamics of activity in the real world, and the dynamics of inner affective life including unreal fantasies and memories. Both are dynamically animated through affects, and normally, there are dynamics unifying them, notably a third types of dynamics, these of meanings, made possible through the semantic system. Finally, all these dynamics can be either fluid, or rigid and static. It is not clear whether Vygotsky sees this as two planes (Fig. 2) (fantasy, activity and meaning-through words as connecting dynamic), or three planes or fields dynamically related (Fig. 3) (activity, word-meaning making, fantasy).

A conception in terms of three planes would allow to understand the “bends” in the thinking of Z. and K: from intention and through language, Z. bends into fantasy, but here affects make her captive and there is no bend back to real activity when the dynamics immobilises; in K, word and meanings plunge in activity, with no bend back to inner life and fantasy as the dynamic stops. A third layer model would also have allowed him to account for intuitions developed in his later years: his openings to the ideas of unconscious dynamics, or at least, to fantasies in which affects are fluid. Also, it seems that Vygotsky remains unable to fully account for the double binding of affects and thinking, and thinking and activity. I would dare to suggest that for this, he would have needed to develop models that includes more layers, or more dimensions. Doing so, he could perhaps have connected his earlier intuition that thought is motivated with the importance of affects, together with the other movement by which experiences can turn into thought and backwards. But enough speculation; let me come back to my line of thought.

## Keeping the Fire

The proposition of following the water semantic field in Lev Vygotsky’s work through the *Notebooks* is a way to acknowledge the importance he himself gave to words and metaphors that shape ideas through language. His deep acquaintance with the arts also led him to use many symbolic resources when writing his *Notebooks* and reasoning scientifically. In this very dialogical mode of expression, I identified a recurrent motive related to water, expressed around three main meaning-complexes: water and its decomposition in hydrogen and oxygen; water as what extinguishes fire; and water as circulating from clouds to the floor through rain and evaporating back into clouds.

Following these semantic fields, I saw first occurrences of water in Vygotsky’s early writing; used in the literal sense, borrowed from literature or the Bible, they seem to have no significance in themselves. However, the fact is that most of the motives they suggest will come back later in Vygotsky’s writing: vivid images, even when their origin is biblical or poetical – water tanks and extinguished fires – seem to remain material mobilised when thinking theoretically, a mode of thinking that demands imagination, free association, and metaphors to be sustained creatively. Following Vygotsky’s early writings, the *Psychology of art* probably also gave him another series of metaphors to hold on; it was also the book in which he developed a core theoretical intuition: that of the two planes of realisation of language for the reader of arts, and the importance of the dynamic tensions between them (Zittoun & Stenner, 2021).

**Fig. 4:**
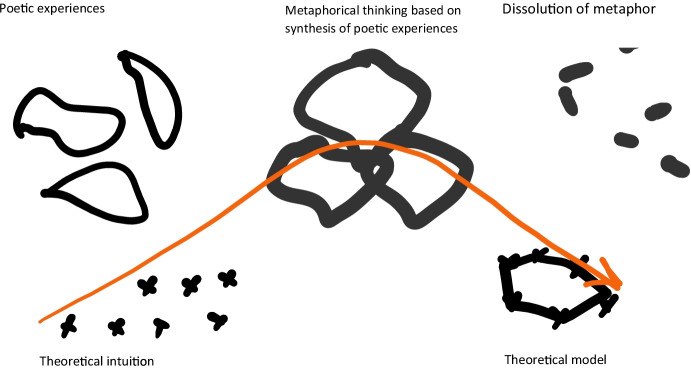
The trajectory of metaphor in theoretical thinking

In 1928, Vygotsky became fully a psychologist, and he attacked the difficult debates of his time: the relation between affects and thinking, and language and thinking. Here, it is important to remind that he came to this idea also through his dialectical training, by which he tends to consider the unity of the opposite, the synthesis of two elements when he approaches a problem (Van der Veer & Valsiner, 1993a). For the problem of thinking and speech, I believe that the image of the cycle between water, clouds and rain, perhaps inspired by his many poetic or political readings, which I have called the meteorological metaphor, played a key-role in Vygotsky’s thinking. My hypothesis is that it supported the transformation of a vague intuition, made conceptually visible, into a plausible theoretical model, that would combine the idea of two planes of realisation, with a demonstration of the dynamics that unite them. As I tried to show, the metaphor lost its importance in Vygotsky’s later writings, as he now could name these processes conceptually, and formulate a theory of inner speech, where thinking is process, and in which signs and language enable generalisation, and the materialisation of ideas into language (see Fig. 4). However, the metaphor sketched by Vygotsky was not fully theoretically explored; as pointed out by Valsiner and Van der Veer, Vygotsky did not fully integrate the importance of motives in thinking (Van der Veer & Valsiner, 1993b, p. 370) – the role of the wind of thinking.

As corollary of this proposition, I can add that the trajectory of a metaphor just highlighted also corresponds to Vygotsky theory of the cultural mediation of higher thinking processes: it is through language and other semiotic forms, including these borrowed from the arts, that we can give shape to our thinking and bring it to higher levels; there, even these linguistic forms may lose their substance, and we may achieve abstract and generalise thinking. In any case, if we can then develop new concepts, we have changed our relationship to the world.

After the dissolution of the meteorological metaphor, Vygotsky continued to work with two-planes models, and although he came to admit the importance of unconscious dynamics as proposed by Freud (and against his earlier critical stance in the *Psychology of art*), my impression is that he could not fully integrate it. The final pages of the *Notebooks* are in that sense very moving: now feeble and ill, Vygotsky tries to reason and articulate his clinical observations and new intuitions, such as the fluidity-rigidity of thinking, with the theoretical system he had built. He appears desperately looking for a way to propose a creative synthesis, yet he seems to lack the elements or the perspective that would enable him to do so. It will not ever be possible to know what exactly Vygotsky had in mind, but he seems to show us the elements of the equation he wishes to solve: looking at people that suffer from a disbalance between affects, language, thinking, meaning, and activity, Vygotsky was trying to design the model of mind that would enable us to account how these dynamics are systemically combined when we do think well enough. My intuition is that the formulation of this model was doubly limited by a way of thinking dialectically through two main layers or fields, and by the underpinning metaphor of water and its circulation. Perhaps, had he had more time, Vygotsky could have found in his wide poetic experience a more powerful symbolic resource to bring his theory to a next degree of integration.

Certainly, learning from Vygotsky’s way of working, his capacity and freedom to follow his intuitions through metaphorical thinking, his incredible capacity to think through a wide diversity of material, his dialogical engagement with colleagues and authors from the past and present, we may also find inspiration to address the most fundamental issues of psychology, still at the heart of contemporary psychology.

## Footnotes

1 In Zavershneva & Van der Veer (2018, p. 210).

2 Here and everywhere in the text, emphases in quotes of Vygotsky are original.

3 “*Psychology is the science of the person* (not of behavior and not of mental phenomena, since it does not study any particular phenomena, but of the soul, understood socially – a return to rational psychology” (Zavershneva & Van der Veer, 2018, p. 79).

4 The Experimental Defectological Institute (EDI) of Narkompros was created in 1929 and directed by Vygotsky from its creation to his death (Zavershneva & Van der Veer, 2018, p.167, note 3).

5 Note here the interesting reflection on the role of symbolic resources in creating a plane of fantasy which may replace a frustrating plane of engagement in real life, when Vygotsky describes one of the children as “hyperaesthetic and sensitive”: he is of no use at home, compensates at school – yet he has a passion for cinema: “Bad children and their role: the *passion for cinema*. After a flogging, the antisocial act disappears. *The cinema is the most typical compensation. That he goes to the cinema and was silent at school is connected*. ‘My brother is an ape, my father is a swine’”. (Vygotsky in Zavershneva & Van der Veer, 2018, p. 188).

6 I do not know why Lenin is mentioned here, and it is not commented by Zavershneva and Van der Veer; I however wondered how much this is related the analogy Vygotsky attributes to Hegel in 1926 (Water-ice-steam).
